# Development of sentinel node biopsy, ROLL and IART in early breast cancer at the European Institute of Oncology, Milan (IEO)

**DOI:** 10.3332/ecancer.2017.744

**Published:** 2017-06-08

**Authors:** Giovanni Paganelli

**Affiliations:** Department of High Technology, Nuclear Medicine and Radiometabolic Unit, Istituto Scientifico Romagnolo per lo Studio e la Cura dei Tumori (IRST) IRCCS, Meldola, Italy; * Former Director of the Division of Nuclear Medicine, European Institute of Oncology, Milan.

**Keywords:** sentinel node, lymphoscintigraphy, ROLL, IART

## Abstract

The problem of unnecessary axillary clearance in many patients with early breast cancer was certainly a major issue at IEO when we started working with Prof. Umberto Veronesi in 1994. At that time, axillary dissection in EBC was offered to all patients and this procedure was often hotly debated during our multidisciplinary breast cancer meetings. The question as to whether we could avoid axillary dissection by using PET scans or other nuclear medicine methods was frequently asked by Veronesi. This eventually prompted us to investigate whether, as for melanoma patients, the sentinel node biopsy (SNB) approach could reliably be applied to breast cancer. In December 1995, we proposed a new lymphoscintigraphy protocol to detect the SN in early breast cancer patients to our Ethic Committee, and it was approved. The pilot study was published in 1997 and after only 6 years, the first randomised trial comparing SNB and axillary clearance in breast cancer patients was published. During the pilot study, we optimised the lymphoscintigraphy technique by comparing different radiotracers and different injection modalities. Following the observation that the majority of the radiocolloids injected into the tumour did not migrate to lymph nodes, a new method called ROLL or Radio-guided Occult Lesion Localisation was developed for the localisation of non-palpable breast lesions. This technique was introduced into clinical practice at the European Institute of Oncology in 1996. Several studies showed that the ROLL procedure enabled the surgeon to remove non-palpable breast lesions easily and accurately, overcoming some disadvantages of other methods such as wire-guided localisation (WGL). In addition to SNB and ROLL, other protocols such as the IART (intraoperative avidination for radionuclide therapy)-ARTHE (avidinated radiotherapy) procedure were developed at the IEO Nuclear Medicine Division during the period 1994–2013. I remember that time as the most professionally productive of my career and it would not have been possible without the help, suggestions and encouragement given to me by Umberto Veronesi.

## Introduction

The most important goal of modern surgical oncology is to utilise the least aggressive methods while maintaining radicalism. That is perhaps the main lesson that all of us who had the pleasure and honour of working alongside Umberto Veronesi received. Sentinel lymph node biopsy (SNB) and radioguided occult lesion localisation (ROLL) of non-palpable tumours are the logical consequence of this lesson. SNB and ROLL represent an important contribution of nuclear medicine to the management of early breast cancer. There is no doubt that the introduction of these techniques in the clinical setting has reduced the morbidity and improved patient quality of life.

The concept of ‘sentinel lymph node’ is connected to the notion that, in the majority of patients, metastatic spread of cancer through the lymphatic system follows a unidirectional, orderly and predictable pattern [1, 2]. On the basis of this assumption, the histological evaluation of the ‘sentinel node’, which is the first lymph node that drains the lymph directly from the primary tumour, allows us to exclude the presence of malignant cells in other lymph nodes.

During the 1990s, the concept of the sentinel lymph node and its possible impact on the surgical treatment of cancer demonstrated its potential in the clinical setting [3, 4]. The use of a vital dye (blue dye), administered subdermally in the region surrounding the lesion, was applied to various types of solid tumours such as melanoma and breast cancer. However, at IEO, the low success rate of the blue dye method led us to further refine lymphoscintigraphy using different compounds with the aim of finding a more reliable method for SNB. This research was begun at our Institute in 1995 and the pilot studies using human serum albumin (HSA) nanocolloid were published in 1997 and 1998 [5, 6]. The first randomised trial comparing total axillary dissection with SNB was then conducted by Veronesi *et al* [7], 516 patients with breast cancer < 2 cm randomised to each of these groups. The IEO study was designed to recruit more than 1000 patients but after preliminary results were published, patients refused to continue randomisation. In 2000, SNB became the standard technique at IEO to stage lymph nodes in patients with early breast cancer and clinically negative axilla. The rest of the world followed rapidly.

In 1996, in addition to the SN study for the optimisation of the lymphoscintigraphy technique, a new method for the localisation of non-palpable breast lesion known as ROLL, indicating ‘radioguided occult lesion localisation’ was also developed. The idea for ROLL came about almost by accident during a coffee break with my friend Alberto Luini (breast surgeon) who asked me if we could find a method similar to that of SN for non-palpable tumours. I put on my thinking cap and Alberto and I soon presented an idea to Umberto Veronesi who suggested using the acronym ROLL. A few days later we began to use this new technique and have since never used any other methods to localise non-palpable lesions in breast cancer [8].

The IART-ARTHE technique is similar to ROLL but with therapeutic intent. IART stands for intraoperative avidination for radionuclide therapy and is performed during surgery. ARTHE refers to avidinated radiotherapy and is used for non-palpable tumours. These protocols are still ongoing and they were the last projects that Prof. Veronesi and I designed together.

The present article reports some of the more important aspects of nuclear medicine relating to the development of SN, ROLL and avidin–biotin pre-targeting techniques.

## Methodological aspects of lymphoscintigraphy

Sentinel lymph node localisation and biopsy (SLNB) represents the ‘standard of care’ for the assessment of axillary lymph nodes in patients with early breast cancer. Although this procedure has completely replaced axillary lymph node dissection (ALND), there is still no general consensus on various methodological aspects of the sentinel lymph node procedure [9]. There are some controversies regarding the type of tracer to be used (different in the US and EU), injection method, type of images to acquire (planar, SPECT) and the subsequent localisation in the operating theatre with a gamma probe.

## Radiotracers

The perfect radiopharmaceutical for sentinel lymph node biopsy should easily drain from the administration area to the first node, accumulating preferentially in one or two nodes and not drainage towards subsequent lymph node stations. In our experience, the ideal tracer is composed of particles between 100 and 200 nm in size in order to obtain the best compromise between speed of drainage and accumulation in the sentinel node. In fact, colloidal tracer particles < 50 nm drain rapidly from lymphatic vessels and move into second- and third-level lymph nodes [6]. Unfortunately, tracer colloidal particles with a diameter of 100–200 nm are not commercially available. Many European researchers use human serum albumin particles with diameters of 40–100 nm (95% < 80 µm), whereas technetium-labelled sulphur and antimony trisulphide are the most widely used radiopharmaceuticals in the United States and Australia/Canada, respectively [10].

In our first series of 240 consecutive patients, the mean number of lymph nodes visualised using radiocolloid particles of 15 and 50 nm was 2.1, 1.6 for tracer particles up to 80 nm, and 1.3 for larger particles [6].

## Injection

Several studies have been carried out to compare the results obtained with the various methods of injection. The different methods proposed can be divided into two main categories: deep injection, that is, intratumoral or peritumoral, and superficial injection, that is, intradermal, subcutaneous, or periareolar [11]. In our experience [6], no significant differences in the identification rate of sentinel nodes has ever been observed using either intradermal or peritumoral injection. The only difference worth reporting is a delay in the visualisation of the sentinel lymph node when using peritumoral administration.

We believe that both injection techniques are valid and often complementary; we currently prefer the hypodermic injection for superficial tumours and peritumoral administration for deep tumours.

## Imaging

In general, a large field gamma camera is used to identify the sentinel node by lymphoscintigraphy. The patient lies in a supine position, with the arm on the side of the intervention raised earlier the head to allow positioning of the gamma camera as close as possible to the axilla. Once the lymph node is visualised, the skin projection of the sentinel lymph node is marked on the axilla using a permanent marker. The added value of SPECT/CT for the visualisation of sentinel nodes has yet to be confirmed.

## Intraoperative gamma probe counting

The intraoperative localisation of sentinel nodes involves the use of a gamma probe sensor wrapped in a sterile sheath. The surgeon can remove the primary tumour and then visualise the primary tumour, or vice versa. When the primary tumour is in the upper outer quadrant, a single incision may be sufficient to remove the tumour and the sentinel node. Once the lymph node has been localised and excised, it is important to check for further areas of tracer accumulation to rule out the presence of other involved lymph nodes. The complete removal of the sentinel node(s) is confirmed by the reduction of the count rate in the axilla to background levels.

The SLNB procedure has undergone several changes and improvements, since it was first developed and is now routinely performed in situations that were considered as contraindications only a few years ago.

## ROLL

The evolution of imaging techniques and the option for using reliable and effective screening tests have greatly facilitated early diagnosis, leading to the identification of smaller and smaller malignant lesions. This is especially true in the area of breast cancer, where clinically occult lesions are diagnosed with increasing frequency and now represent about 25–35% of all breast cancers diagnosed in developed countries [12].

Wire-guided localisation (WGL) has long been considered the standard technique for the localisation of non-palpable lesions. Under either ultrasound or stereotactic guidance, a thin, hooked wire is inserted into the lesion and the surgeon uses the wire and standard imaging to identify and remove the lesion. However, many studies have reported a high rate of positive margins after wire localisation [13], making it necessary to re-operate and increasing the incidence of local recurrence.

In the ROLL technique, a radioactive tracer (Tc-99MAA) is injected into the tumour under stereotactic or ultrasound guidance on the day before surgery. A hand-held gamma probe (as for sentinel lymph node biopsy) is then used to guide intra-operative identification of the tumour and surgical resection [14]. Several studies have reported fewer positive margin, lower re-operation rates and smaller surgical excision volumes [15].

## The IART approach

The experience gained by our Institute with the ROLL technique and the use of radionuclide therapy with the avidin–biotin pre-targeting system in brain tumours [16, 17] led us to develop a new approach called I.A.R.T. (intra-operative avidination for radionuclide therapy) which is capable of controlling recurrence as effectively as external beam radiotherapy (EBRT).

The IART procedure consists of a first step where the surgeon intra-operatively injects avidin directly into the tumour bed and a second step the following day of an intravenous injection of ^90^Y/radiolabelled biotin [18]. The intra-operatively injected avidin percolates through the tissue of the index quadrant and is drained by locoregional lymph nodes. Its positive electric charge (pI 10.7) and the inflammatory reaction after surgery results in avidin being retained at the site of the injection for several days, constituting a sort of new ‘artificial receptor’ only expressed in the breast area which is capable of homing radioactive biotin and of delivering a dose as high as 20–40 Gy. IART takes advantage of the high affinity between avidin and biotin and mimics the well-known model of radiometabolic treatment with ^131^I in differentiated thyroid carcinoma.

Two clinical studies were conducted on IART at IEO: a phase-I dosimetric study involving 11 patients [18] and a phase-II dose range study of 35 patients [19]. Detailed dosimetry data from both studies show that intravenous administration of a dose of 3.7 GBq 90Y- DOTA-biotin after intra-operative injection of 100 mg of avidin in the tumour bed provided an absorbed dose of 19.5 ± 4.0 Gy, corresponding to a biological effective dose (BED) of 21.2 ± 4.3 Gy in the tumour bed. The results from these studies confirmed that IART was safe and capable of delivering, one day after surgery, a 20 Gy dose in women undergoing surgery for breast cancer. IART was also well accepted by all patients as it did not alter their quality of life.

Based on the positive results of the phase-II trial carried out at IEO, we are currently planning to conduct a phase-III trial at IRST on a large number of breast cancer patients (around 1000 per arm) who are candidates for EBRT after surgery. The main aim of the study will be to demonstrate that IART is not inferior to conventional EBRT in terms of preventing local recurrence after conservative breast surgery. If the outcome of this randomised trial is positive, IART could be proposed as a new procedure for breast irradiation, offering numerous practical advantages over other methods. An important advantage is its potential applicability to all breast cancer patients scheduled for conservative surgery, regardless of tumour location, size or multifocality. When using IART, the irradiation field is accurately identified by the surgeon who knows exactly where the tumour is located and therefore injects avidin under visual control directly into the tumour bed.

Another advantage of IART is it does not require a dedicated linear accelerator or other sophisticated devices. Furthermore, it is a procedure that can be used in any hospital where conservative breast surgery pioneered by Umberto Veronesi is performed [20].

## ARTHE: avidinated radionuclide therapy

Non-palpable breast lesions (NPBL) represent about 30% of the overall number of breast lesions requiring surgery. The vacuum-assisted breast biopsy (VABB) is a procedure for needle breast biopsy that is highly effective for diagnosing NPBLs and has minimal side effects. The specimen volume obtained through this procedure is sufficient to replace the material obtained from diagnostic surgical excisional biopsy and frozen section intra-operative examination.

NPBLs submitted to VABB include microcalcifications, asymmetrically dense areas, and circumscribed and spiculated masses < 15 mm in diameter. A complete excision of the lesion is sometimes possible using this method, making it a valid alternative to the excisional biopsy for benign lesions < 15 mm.

A preliminary analysis of results on VABB procedures performed at IEO under stereotactic guidance for microcalcification clusters revealed that, of 781 cases positive for cancerous lesions (*in situ* or infiltrating carcinomas), 559 (71.5%) had residual disease following the procedure, identified by X-ray. The same imaging confirmed the complete removal of the microcalcifications in the remaining 222 cases. In this group, histological analysis of the surgical specimen confirmed a disease-free status in 31.5% of patients (*E. Cassano personal communication*), whereas 68.5% of cases had microscopic residual disease in the area of the VABB specimen withdrawal site, despite the negative mammogram. Our idea was to inject a radionuclide compound into the lesion site following the VABB procedure to eradicate residual tumour cells. This technique, which we called ARTHE (avidinated radionuclide therapy), was obviously based on our ROLL experience in localising NPBLs. In fact, previous studies on IART showed that, when injected into breast tissue, avidin molecules remain at the injection site, permitting the uptake of intravenously administered radioactive biotin.

In the ARTHE technique, avidin is injected under ultrasound guidance into the site of the non-palpable lesion a few days after the VABB procedure. Clearly, the patients enrolled in ARTHE studies are submitted to standard surgical procedures and the efficacy of the approach is assessed by pathological examination of the removed tissue. In our opinion, ARTHE radionuclide therapy could increase the rate of patients who are histologically confirmed as disease-free after excisional biopsy. The approach could also reduce the need for surgical intervention and decrease surgery-related morbidity, thus improving the quality of life of patients. Moreover, waiting lists and healthcare costs could be substantially reduced.

## Conclusions

**Figure 1.**
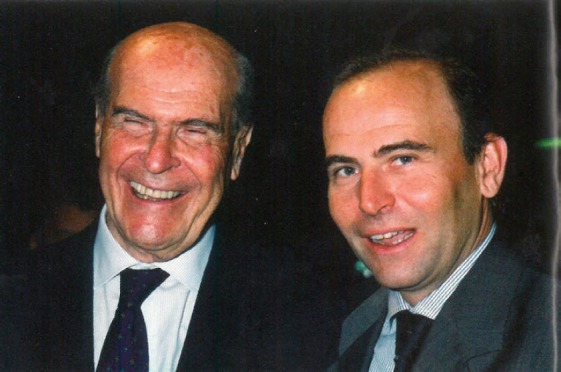
Giovanni Paganelli and Umberto Veronesi in 2001 when the randomised study on the sentinel lymph node was ongoing.

In this article, I recalled some of the most important moments of my work experience alongside Umberto Veronesi (Figure 1). Today, the findings from some of these studies have entered routine clinical practice, and this gives me immense personal satisfaction. Conversely, techniques, such as IART and ARTHE, are still undergoing evaluation, and much remains to be done before they become standard procedures. It is perhaps logical that such innovative methods go a little against the grain and are not immediately welcomed by the scientific community. This was the case with SNB and ROLL in that both approaches clashed somewhat with standard procedures. The challenge was to believe that things could change for the better, but only by proving hypotheses scientifically. The common thread linking SNB, ROLL, and IART is the search for a new, effective, non-invasive tool that fully respects the dignity and quality of life of our patients. This is the message that Umberto Veronesi conveyed to those who were fortunate enough to be part of his team. I am immensely grateful and can only hope that I am equally capable of transmitting such insight to the physicians I work with.

Thank you, Prof. Veronesi.

Thanks, Umberto.
